# The impact of prematurity and maternal socioeconomic status and education level on achievement-test scores up to 8^th^ grade

**DOI:** 10.1371/journal.pone.0198083

**Published:** 2018-05-31

**Authors:** Nahed O. ElHassan, Shasha Bai, Neal Gibson, Greg Holland, James M. Robbins, Jeffrey R. Kaiser

**Affiliations:** 1 Department of Pediatrics, Division of Neonatology, University of Arkansas for Medical Sciences, Little Rock, Arkansas, United States of America; 2 Department of Pediatrics, Division of Biostatistics, University of Arkansas for Medical Sciences, Little Rock, Arkansas, United States of America; 3 Arkansas Research Center, University of Central Arkansas, Conway, Arkansas, United States of America; 4 Department of Pediatrics, Division of Child Health Services Research, University of Arkansas for Medical Sciences, Little Rock, Arkansas, United States of America; 5 Department of Psychiatry, University of Arkansas for Medical Sciences, Little Rock, Arkansas, United States of America; 6 Department of Pediatrics, Division of Neonatology, Milton S. Hershey Medical Center, Hershey, Pennsylvania, United States of America; 7 Department of Obstetrics and Gynecology, Milton S. Hershey Medical Center, Hershey, Pennsylvania, United States of America; TNO, NETHERLANDS

## Abstract

**Background:**

The relative influence of prematurity vs. maternal social factors (socioeconomic status and education level) on academic performance has rarely been examined.

**Objective:**

To examine the impact of prematurity and maternal social factors on academic performance from 3^rd^ through 8^th^ grade.

**Methods:**

We conducted a retrospective cohort study of infants born in 1998 at the University of Arkansas for Medical Sciences. The study sample included 58 extremely low gestational age newborns (ELGANs, 23‒<28 weeks), 171 preterm (≥28‒<34 weeks), 228 late preterm (≥34‒<37 weeks), and 967 term ((≥37‒<42 weeks) infants. Neonatal and maternal variables were collected including maternal insurance status (proxy measure for socioeconomic status) and education level. The primary outcomes were literacy and mathematics achievement-test scores from 3^rd^ through 8^th^ grade. Linear mixed models were used to identify significant predictors of academic performance. All two-way interactions between grade level, gestational-age (GA) groups, and social factors were tested for statistical significance.

**Results:**

Prematurity, social factors, gender, race, gravidity, and Apgar score at one minute were critical determinants of academic performance. Favorable social factors were associated with a significant increase in both literacy and mathematic scores, while prematurity was associated with a significant decrease in mathematic scores. Examination of GA categories and social factors interaction suggested that the impact of social factors on test scores was similar for all GA groups. Furthermore, the impact of social factors varied from grade to grade for literacy, while the influence of either GA groups or social factors was constant across grades for mathematics. For example, an ELGAN with favorable social factors had a predicted literacy score 104.1 (P <.001), 98.2 (P <.001), and 76.4 (P <.01) points higher than an otherwise similar disadvantaged term infant at grades 3, 5, and 8, respectively. The difference in their predicted mathematic scores was 33.4 points for all grades (P <.05).

**Conclusion:**

While there were significant deficits in academic performance for ELGANs compared to PT, LPT, and term infants, the deficit could be offset by higher SES and better-educated mothers. These favorable social factors were critical to a child’s academic achievement. The role of socioeconomic factors should be incorporated in discussions on outcome with families of preterm infants.

## Introduction

Academic outcomes are shaped by many factors including the degree of prematurity [1–3] and the social environment [4]. Maternal socioeconomic status (SES) and education level have been repeatedly recognized as critical social determinants of neonatal outcomes [4]. Social advantadges strongly predict gains in cognitive scores in the preschool years [5], while adverse social status are associated with lower educational attainment [4,6]. Although children born prematurely are at increased risk for academic underachievement [1–3,7,8], they are not uniformly committed to poor academic performance, as some born at the limits of viability can be gifted students at school age [2].

Most school outcome studies reported school completion [9], relied on subjective parental recall of neonatal characteristics [10] or potentially biased parental or teacher appraisal of a student’s performance [3,11,12], or focused on placement in special education [13,14]. In addition, academic performance can be evaluated by aptitude tests or achievement-tests. Previous studies largely relied on aptitude test results which are primarily designed to predict a child’s ability to learn new skills [15,16]. In contrast, standardized school achievement tests are valid curriculum-based measures rather intended to reflect what a child has actually learned [17]. Performance on achievement tests was recently proposed to be closely aligned with “real-world” outcomes such as high-school graduation, college attendance, and long-term adult success [18]. Although some recent academic outcome studies included standardized achievement tests [1,2,7,19], none explicitly evaluated the relative contribution of prematurity and maternal social factors on test scores.

We investigated the relative influence of prematurity and maternal SES and education level on achievement-test score trajectory from 3^rd^ through 8^th^ grade (8–13 years of age) from all infants (23–42 weeks’ gestation) born in 1998 at the University of Arkansas for Medical Sciences (UAMS). We speculated that an individual’s academic performance during childhood and early adolescence is only partially determined by the degree of prematurity and that performance deficits due to prematurity could be offset by having a mother with favorable social factors (high SES and advanced education level). Our *a priori* hypothesis was that favorable maternal social factors (high SES and advanced education level) have greater influence on achievement-test scores than the degree of prematurity.

## Materials and methods

The study received University of Arkansas for Medical Sciences (UAMS) Institutional Review Board approval and waiver of consent. Health Insurance Portability and Accountability Act (HIPAA) and Family Educational Rights and Privacy Act (FERPA) waivers were also obtained.

All surviving infants born at UAMS in 1998 were considered for study inclusion. Infants with major congenital anomalies or chromosomal abnormalities were excluded. Four gestational age (GA) groups were defined: extremely low gestation newborns (ELGANs, 23 to <28 weeks’) [20], preterm (PT, ≥28 to <34 weeks’) [21], late preterm (LPT, ≥34 to <37 weeks’) [21], and term (≥37 to <42 weeks’) infants [22]. Data abstracted from the newborn medical record included name, birth date, birth weight, estimated GA, size for GA, race, gender, singleton or multiple gestation, delivery mode, Apgar scores, meconium at delivery, length of stay, and brain injury (mild: grades 1–2 intraventricular hemorrhage; severe: grades 3–4 intraventricular hemorrhage, periventricular leukomalacia, and/or ventriculomegaly). Maternal data was collected by extensive review of each maternal medical record and included name, age, gravidity, medical conditions, prenatal care, smoking, substance abuse, and maternal education level and insurance status. Insurance status, collected at the time of maternal hospital admission, was used as a proxy measure for SES [23]. Medicaid, no insurance, and “self-pay” were deemed low SES while private insurance was considered high SES. Less than a high school education or high school graduation was deemed low education level, while postsecondary education was considered high education level. These maternal social factors (SES and education level) were used since they were previously identified separately as significant predictors of neonatal outcomes [4,24]. We combined them, as follows, in four strata since children, in any social setting, will be exposed to both variables rather than either one alone: low SES and low education (LL), low SES and high education (LH), high SES and low education (HL), and high SES and high education (HH). Each stratum evaluates the impact of both maternal SES and education level and their interaction and therefore, the analyses in this manuscript do not allow for the evaluation of either social factor alone on achievement-test scores. Data were stored in Research Electronic Data Capture (REDCap) (1UL1RR029884) hosted at the UAMS Translational Research Institute [25].

### Arkansas Department of Health and Department of Education

The dataset was securely transmitted to the Arkansas Department of Health where social security numbers were added; this dataset was then securely transmitted to the Arkansas Research Center of the Arkansas Department of Education. Newborn name, birth date, and social security number were conservatively matched with comparable identifiers in the Arkansas Department of Education database [26]. The Arkansas Department of Education database includes scores and proficiency designations from Arkansas public school students who take the annually administered Arkansas Augmented Benchmark Examination tests in literacy and mathematics (grades 3–8). Achievement-test scores were based on scaled scores of 0 to 1000 and were assigned according to the percentage of correct answers. Scores designated as proficient or advanced (“proficient”) represented performance at or above grade level, while scores designated “non-proficient” represented performance below grade level [26]. The proficiency threshold (i.e., the specific achievement-test score), set by the Arkansas Department of Education, increased for each advancing grade level. Students who were home schooled, attended private schools, moved out of Arkansas, died after hospital discharge and before fourth grade, or who had significant cognitive disabilities (n = 11), did not take the Benchmark Examination and were excluded from the analysis.

The final data file, including all UAMS-born participants who were successfully matched to their student achievement-test scores, was encrypted and transmitted back to the UAMS researchers [26]. A successful match was defined as a newborn who had test information available at any grade. The primary outcomes were 3^rd^ through 8^th^ grade literacy and mathematics achievement-tests scores and their proficiency designation.

### Statistical analysis

Summary statistics including mean and standard deviation, median and interquartile range for continuous variables, or frequency and percentage for categorical variables, were determined for each GA group. Score availability and percentages of literacy and mathematics proficiency were evaluated for each grade by GA group.

A key issue in the analysis of longitudinal data is that outcomes measured repeatedly within the same subject tend to be correlated and this correlation structure needs to be taken into account [27]. Linear mixed models were used in preference to standard linear regressions since mixed models assume that measurements from a single subject share a set of unobserved random effects which are used to generate an association structure between the repeated measurements [27].

#### Predictive models

A univariate linear mixed-effect model was fit using the neonatal and maternal variables to predict test scores. The estimated coefficients and 95% confidence intervals (CIs) from the univariate analysis for potential variables affecting test scores were then determined. Predictor variables included in the multivariate model were selected if the p-value from the univariate analysis was <0.10 and/or if it was deemed clinically relevant (determined *a priori*). Backward stepwise selection was used to determine the final set of significant variables. Collinearity was examined using a variance inflation factor. The effect of each variable on test scores was computed. To study the time-varying effect of prematurity and social factors, two-way interactions between grade level, GA groups, and social factors were tested for statistical significance. Interaction is tested by adding a term to the model in which the two predictor variables are multiplied. Models were fit separately for literacy and mathematics. Predicted achievement-test scores were computed from the final mixed-effect models. Pairwise comparisons of scores between different combinations of GA group and social factors were explored using contrasts. Statistical analysis was performed with Stata software (version 14.1; StataCorp LP, College Station, TX). P-values <0.05 were considered statistically significant.

## Results

Descriptive characteristics of the study sample by GA group are summarized in Table 1. Newborns unmatched to achievement-test scores were similar to matched infants [26]. Eighty-four percent (1195 of 1424) of the matched cohort were LPT and term infants. Overall, 79.6% (1134 of 1424) were appropriate size for GA, 94.6% (1347 of 1424) were black or white, and 50.5% (719 of 1424) were male. Five-minute Apgar scores ≥7 were reported in 93.3% (1313 of 1407) of infants and severe brain injury was described in only 1.3% (18 of 1424) of patients. Fifty-six percent (776 of 1379) of mothers were 20–29 years old, 66.1% (939 of 1420) were multigravida, and 94.9% (1352 of 1424) were non-diabetic. Eighty-one percent (1092 of 1345) of mothers had low SES, while 25.4% (342 of 1345) had education beyond high school. The proportion of white and black ELGANs infants with maternal social strata (LL) was similar (3.4% vs. 3.8%) (S1 and S2 Tables). The proportion of ELGANs who had disadvantaged mothers (social strata-LL) was lower than term infants who had mothers with similar social strata. In addition, a larger percentage of black infants compared to white infants had mothers with social strata (LL) (72.6% vs. 61.7%) (S1 and S2 Tables).

**Table 1:** Patient characteristics of the final matched cohort.

Variables	ELGAN	PT	LPT	TERM	P^b^	Matched^a^ Total
	(n = 58)	(n = 171)	(n = 228)	(n = 967)		(n = 1424)
**Neonatal**						
**Birth weight**, mean (SD), g	829 (162)	1609 (466)	2609 (548)	3288 (504)	<.001	2877 (859)
**Gestational age**, mean (SD), week	25.8 (1.1)	30.8 (1.7)	35.4 (0.8)	38.9 (1.3)	<.001	36.8 (3.8)
**Size at birth**, n (%)					<.001	
AGA	56 (96.6)	144 (84.2)	172 (75.4)	762 (78.8)		1134 (79.6)
LGA	0 (0)	1 (0.6)	7 (3.1)	86 (8.9)		94 (6.6)
SGA	2 (3.4)	26 (15.2)	49 (21.5)	119 (12.3)		196 (13.8)
**Race**, n (%)					<.001	
Black	30 (51.7)	60 (35.1)	104 (45.6)	471 (48.7)		665 (46.7)
White	28 (48.3)	109 (63.7)	106 (46.5)	439 (45.4)		682 (47.9)
Other	0 (0)	2 (1.2)	18 (7.9)	57 (5.9)		77 (5.4)
**Male**, n (%)	27 (46.6)	89 (52)	111 (48.7)	492 (50.9)	.83	719 (50.5)
**Multiple gestation**, n (%)	19 (32.7)	36 (21.1)	21 (9.2)	8 (0.8)	<.001	84 (5.9)
**Vaginal delivery**, n (%)	28 (48.3)	85 (49.7)	151 (66.2)	715 (73.9)	<.001	979 (68.7)
**Apgar 1 minute, median** [IQR]	4 [3–6]	7 [6–8]	8 [6–9]	8 [7–9]	<.001	8 [7–9]
≥7, n (%)	10 (17.9)	95 (56.5)	167 (73.6)	826 (86)		1098 (77.8)
*n missing*	2	3	1	7		13
**Apgar 5 minutes, median** [IQR]	7 [6–7]	8 [7–8]	9 [8–9]	9 [9–9]	<.001	9 [8–9]
≥7, n (%)	33 (60)	137 (81.5)	209 (92.5)	934 (97.5)		1313 (93.3)
*n missing*	3	3	2	9		17
**Meconium**, n (%)	0 (0)	4 (2.3)	18 (7.9)	116 (12)	<.001	138 (9.7)
**LOS**, median [IQR], days	78.5 [68–89]	26 [17–41]	5 [3–10]	3 [2–3]	<.001	3 [2–6]
**Brain injury**, n (%)					<.001	
None	35 (60.3)	152 (88.9)	228 (100)	967 (100)		1382 (97.0)
Mild	7 (12.1)	17 (9.9)	0 (0)	0 (0)		24 (1.7)
Severe	16 (27.6)	2 (1.2)	0 (0)	0 (0)		18 (1.3)
**Maternal**						
**Maternal age**, mean (SD), years	24.8 (6.7)	24.3 (6.7)	24.8 (6.5)	23.7 (5.9)	.064	24.0 (6.1)
<20, n (%)	10 (18.2)	45 (27.3)	52 (23.6)	249 (26.5)		356 (25.8)
20–29, n (%)	31 (56.4)	91 (55.2)	118 (53.6)	536 (57.1)		776 (56.3)
30–39, n (%)	11 (20)	22 (13.3)	47 (21.4)	139 (14.8)		219 (15.9)
≥40, n (%)	3 (5.5)	7 (4.2)	3 (1.4)	15 (1.6)		28 (2.0)
*n missing*	3	6	8	28		45
**Gravidity**, median [IQR]	2 [1–3]	2 [1–3]	2 [1–4]	2 [1–3]	.006	2 [1–3]
1, n (%)	25 (43.1)	69 (40.4)	65 (28.6)	322 (33.4)		481 (33.9)
2–3, n (%)	25 (43.1)	73 (42.7)	104 (45.8)	465 (48.2)		667 (47.0)
>3, n (%)	8 (13.8)	29 (17)	58 (25.6)	177 (18.4)		272 (19.1)
*n missing*	0	0	1	3		4
**Maternal diabetes**, n (%)					<.001	
Non-diabetic	56 (96.6)	161 (94.2)	202 (88.6)	933 (96.5)		1352 (94.9)
Pre-pregnancy	1 (1.7)	5 (2.9)	18 (7.9)	11 (1.1)		35 (2.5)
Gestational	1 (1.7)	5 (2.9)	8 (3.5)	23 (2.4)		37 (2.6)
**PIH**, n (%)	13 (22.4)	52 (30.4)	76 (33.3)	146 (15.1)	<.001	287 (20.1)
**PROM**, n (%)	29 (50)	75 (43.9)	57 (25)	32 (3.3)	<.001	193 (13.5)
Chorioamnionitis, n (%)	8 (13.8)	20 (11.7)	4 (1.8)	34 (3.5)	<.001	66 (4.6)
**Prenatal care**, n (%)	57 (98.3)	164 (95.9)	221 (96.9)	941 (97.3)	.72	1383 (97.1)
**Smoking**, n (%)	1 (1.7)	13 (7.6)	8 (3.5)	12 (1.2)	<.001	34 (2.4)
**Substance abuse**, n (%)	1 (1.7)	6 (3.5)	8 (3.5)	16 (1.7)	.20	31 (2.2)
**Social factors**, n (%)					.07	
LL	31 (53.5)	103 (61.7)	146 (68.5)	627 (69.1)		907 (67.4)
LH	11 (18.9)	20 (11.9)	28 (13.2)	126 (13.9)		185 (13.7)
HL	7 (12.1)	20 (11.9)	13 (6.1)	56 (6.2)		96 (7.1)
HH	9 (15.5)	24 (14.4)	26 (12.2)	98 (10.8)		157 (11.7)
*n missing*	0	4	15	60		79

Abbreviations: AGA, appropriate for gestational age; ELGAN, extremely low gestation newborn; g, grams; IQR, interquartile range; LGA, large for gestational age; LOS, length of stay; LPT, late preterm; PIH, pregnancy-induced hypertension; PROM, prolonged rupture of membranes; PT, preterm; SD, standard deviation; SGA, small for gestational age; Social factors: LL–Low SES, Low Maternal Education; LH–Low SES, High Maternal Education; HL–High SES, Low Maternal Education; HH–High SES, High Maternal Education.

^a^A match is defined as a newborn who has test information available at any grade from the Arkansas Department of Education database.

^b^P-values obtained by analysis of variance for birth weight, gestational age, and maternal age; Kruskal-Wallis test for Apgar 1 minute, Apgar 5 minutes, LOS and gravidity; and Chi-Square test for all categorical variables.

**Fig 1:**
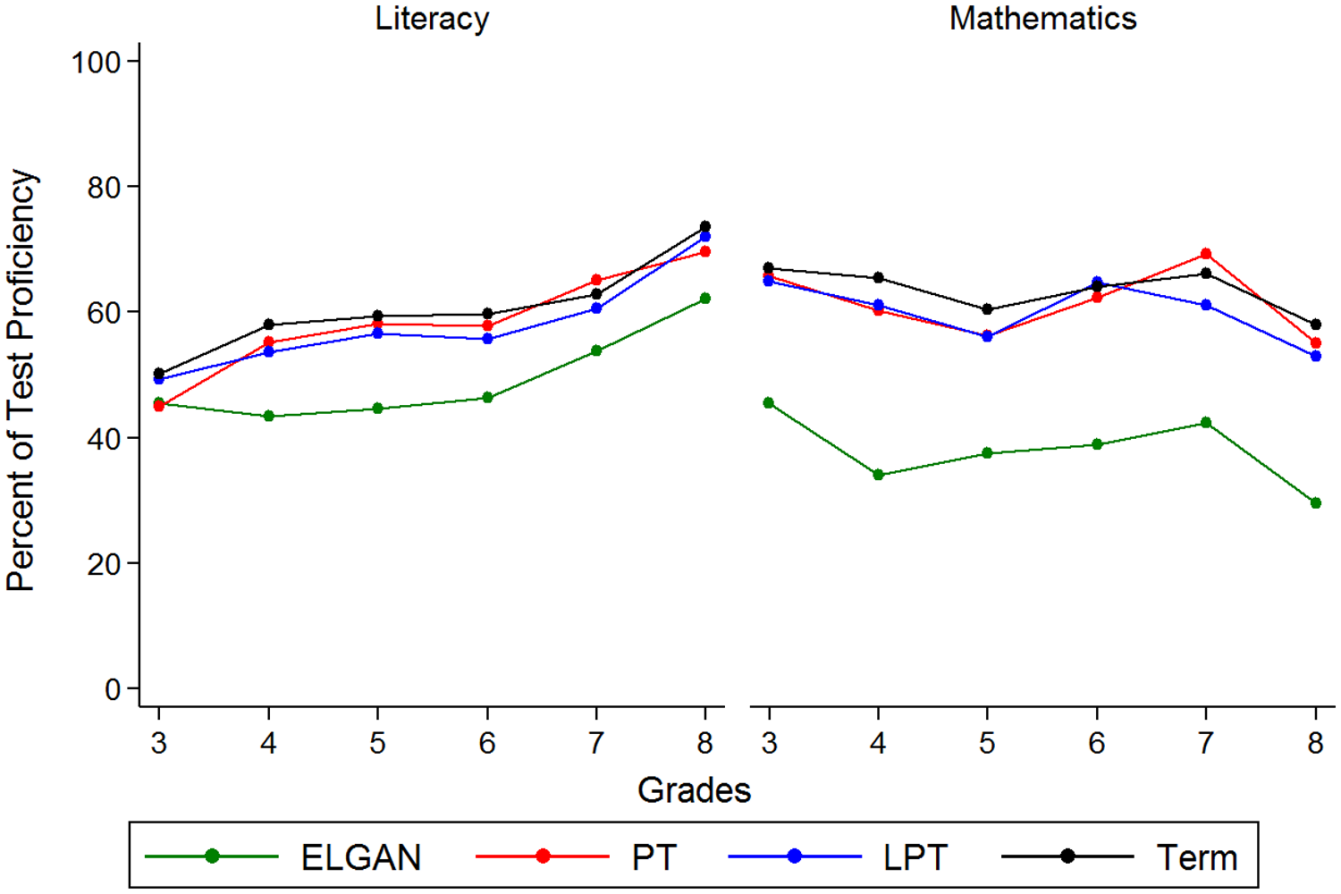
Literacy and mathematics test proficiency by gestational age group and grade level.

Over 90% of newborns were matched to their 3^rd^–7^th^ grade achievement-test scores (S3 Table) Matching was slightly lower (75.9–83.9%) for 8^th^ grade. From 3^rd^–8^th^ grade, the unadjusted ELGAN literacy proficiency rate increased from 46 to 62% and decreased for mathematics from 46 to 30% (Fig 1). Unadjusted PT, LPT, and term infant’s literacy proficiency rates were similar and increased from 45–50% to 70–74%, while mathematics proficiency rates decreased from 65–67% to 53–58%. The proficiency rates for ELGANs (particularly in mathematics) were noticeably lower than the other GA groups.

Achievement-test scores are summarized by GA groups and grade level (S4 Table). Results of the univariate linear mixed models for literacy and mathematics achievement-test scores are shown in S5 Table. Multivariate results are presented in Table 2. Coefficients for each variable represent the estimated mean difference in scores between two infants who differ only on that specific variable. For example, the average decrease in predicted literacy and mathematics scores for ELGANs compared to term infants with similar characteristics was 45.8 and 48.3 points, respectively. Prematurity was associated with a significant decrease in mathematics scores. Male gender was associated with a significant reduction in literacy and mathematics scores, while higher grade, white or other race, Apgar score at 1 minute ≥7, and favorable social factors were associated with increased scores.

**Table 2:** Multivariate linear mixed model for literacy and mathematics scores^a^.

Predictor Variable	Literacy Model			Mathematics Model		
	Coefficient	95% CI	P	Coefficient	95% CI	P
**Grade**			<.001			<.001
3	Ref	Ref		Ref	Ref	
4	107.2	(99.5, 114.9)		58.5	(55.0, 62.0)	
5	160.4	(152.4, 168.4)		90.3	(86.7, 93.9)	
6	192.3	(183.9, 200.7)		139.6	(135.8, 143.3)	
7	237.5	(228.6, 246.5)		168.5	(164.4, 172.5)	
8	300.7	(291.0, 310.5)		175.8	(171.5, 180.2)	
**Gestational age**			.197			<.001
TERM	Ref	Ref		Ref	Ref	
LPT	-6.3	(-28.3, 15.6)		-6.6	(-18.9, 5.8)	
PT	-11.2	(-36.7, 14.3)		-11.0	(-25.3, 3.4)	
ELGAN	-45.8	(-88.0, -3.5)		-48.3	(-72.1, -24.6)	
**Race**			<.001			<.001
Black	Ref	Ref		Ref	Ref	
White	78.9	(62.6, 95.2)		56.8	(47.6, 65.9)	
Other	93.4	(57.9, 129.0)		72.7	(52.7, 92.8)	
**Male**	-79.5	(-94.8, -64.2)	<.001	-13.6	(-22.1, -5.0)	.002
Multiple gestation	-33.2	(-67.7, 1.2)	.058	-17.0	(-36.4, 2.4)	.086
Apgar 1 minute ≥7	33.4	(13.5, 53.4)	.001	17.6	(6.4, 28.8)	.002
**Gravidity**			.002			<.001
1	Ref	Ref		Ref	Ref	
2–3	-22.2	(-39.4, -5.1)		-12.1	(-21.7, -2.5)	
>3	-37.9	(-60.1, -15.7)		-23.2	(-35.6, -10.7)	
**Maternal diabetes**			.223			.069
None	Ref	Ref		Ref	Ref	
Pre-pregnancy	-40.9	(-91.9, 10.1)		-28.9	(-57.5, -0.4)	
Gestational	16.4	(-31, 63.8)		15.1	(-11.5, 41.8)	
**Social factors**			<.001			<.001
LL	Ref	Ref		Ref	Ref	
LH	54.4	(26.1, 82.6)		30.4	(17.6, 43.2)	
HL	79.9	(42.2, 117.7)		20.9	(3.9, 38.0)	
HH	149.9	(119.3, 180.5)		81.7	(67.8, 95.6)	
**Social factors x grade interaction**^b^			<.001			
LH, grade 4	5.3	(-13.5, 24.1)				
LH, grade 5	-21	(-40.5, -1.6)				
LH, grade 6	2.2	(-18.3, 22.6)				
LH, grade 7	-10.2	(-31.9, 11.6)				
LH, grade 8	-14.5	(-38.1, 9.2)				
HL, grade 4	-26.1	(-51, -1.3)				
HL, grade 5	-41.8	(-67.6, -16)				
HL, grade 6	-40.4	(-67.5, -13.4)				
HL, grade 7	-20.3	(-49, 8.4)				
HL, grade 8	-52.6	(-83.4, -21.9)				
HH, grade 4	6.7	(-13.1, 26.6)				
HH, grade 5	-5.9	(-26.4, 14.6)				
HH, grade 6	-3.5	(-25.1, 18.2)				
HH, grade 7	-24.8	(-47.9, -1.8)				
HH, grade 8	-27.8	(-52.7, -2.8)				

Abbreviations: CI, confidence interval; Coef, coefficient; ELGAN, extremely low gestation newborn; LPT, late preterm; PT, preterm; Ref, reference; Social factors: LL–Low SES, Low Maternal Education; LH–Low SES, High Maternal Education; HL–High SES, Low Maternal Education; HH–High SES, High Maternal Education.

^a^The final model includes only the set of variables that are significant at P <.05 or deemed clinically important.

^b^The interaction between social factors and grade was not significant in the mathematics model.

Two-way interactions between grade level, GA groups, and social factors were tested. The interaction between GA categories and social factors was not significant, indicating that the impact of social factors on test scores was similar for all GA groups. The interactions of GA categories or social factors and grade levels were not significant in the mathematics model indicating that the impact of prematurity or social factors was constant across grades. Since the effect of GA categories on predicted literacy scores was not significant, an interaction of prematurity and grade level was not explored. However, the interaction between social factors and grade level was significant in the literacy model implying that the influence of social factors varied across grades. The coefficients of interaction for any social factors and grade level decreased with advancing grades. For example, the average gain in predicted literacy scores secondary to favorable social factors decreased from 149.9 (coefficient [social factors, HH]) at grade 3, to 144 (coefficient [social factors, HH] + coefficient [social factors, HH x grade 5 interaction] = 149.9–5.9) at grade 5, and 122.1 at grade 8.

**Fig 2:**
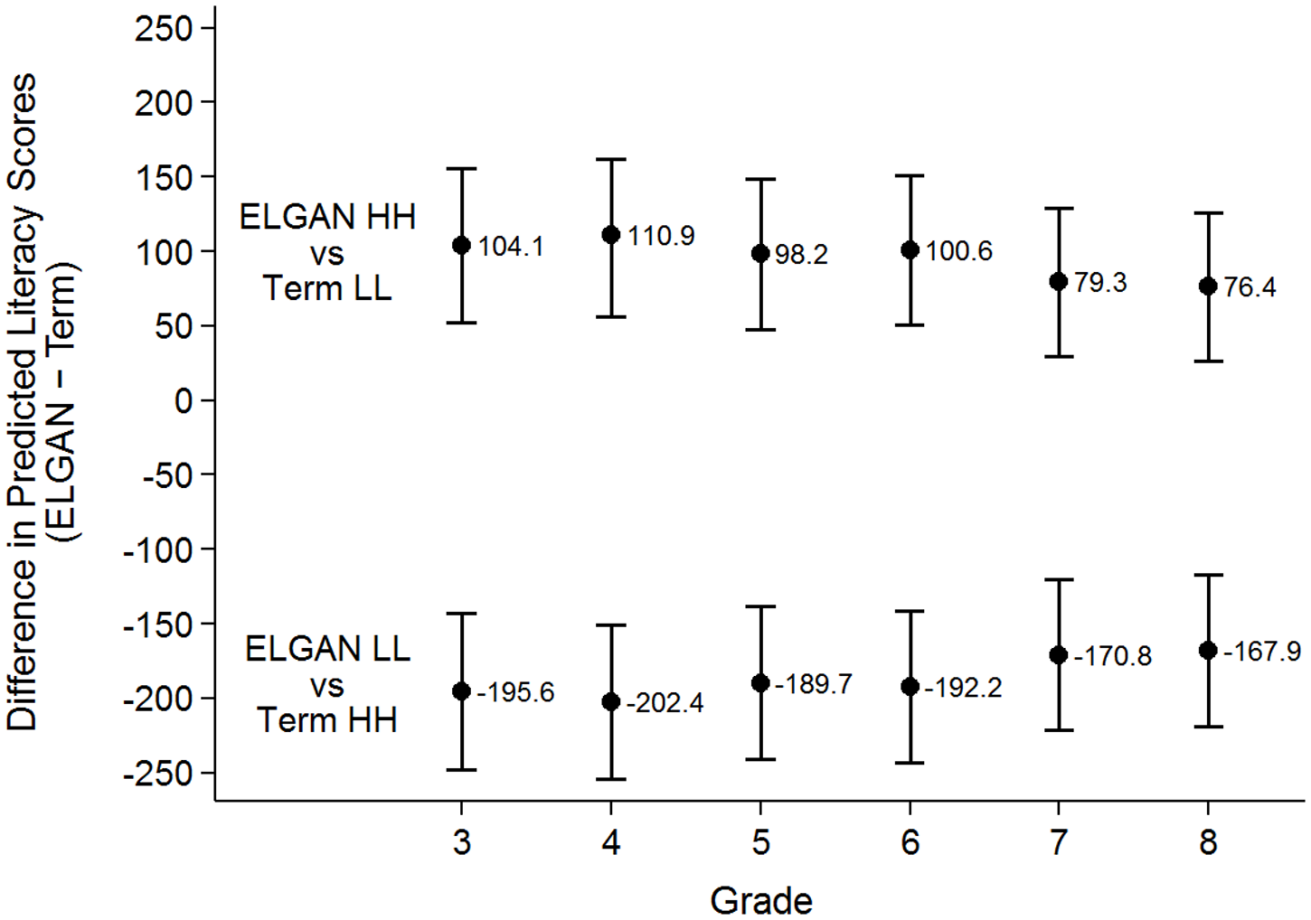
Difference in predicted literacy scores across grades between ELGANs and term infants.

Test scores were lowest in ELGANs, but were similar among LPT, PT, and term infants. The significance of the impact of the different levels of social factors on literacy (Table 3) and mathematics (Table 4) test scores for ELGAN vs. term infants was explored. The calculations were computed at multiple grades for literacy since the influence of social factors varied over time in this model, while a single estimate was applicable to all grades in the mathematics model. Disadvantaged ELGANs performed significantly worse than advantaged (HH) term infants, and term infants with either maternal high SES (HL) or high education (LH). ELGANs with maternal low SES and high education (LH) had comparable predicted literacy scores to disadvantaged (LL) term infants and term infants with maternal high SES and low education (HL). Similarly, ELGANs with maternal high SES and low education (HL) had comparable predicted literacy scores to disadvantaged term infants and term infants with maternal low SES and high education (LH). ELGANs with favorable (HH) social factors had significantly higher predicted literacy scores than disadvantaged (LL) term infants (Table 3 and Fig 2); the difference in their predicted literacy scores decreased with advancing grades. At 3^rd^ grade, the difference was 104.1 (coefficient [GA] + coefficient [social factors, HH] = − 45.8 + 149.9) (P <.001). At 5^th^ grade, it decreased to 98.2 (coefficient [GA] + coefficient [social factors, HH] + coefficient [social factors, HH x grade 5 interaction] = − 45.8 + 149.9–5.9) (P <.001). At 8^th^ grade, it further decreased to 76.4 points (P <.01).

**Table 3:** Difference in predicted literacy scores between ELGANs and term infants at different levels of maternal social factors from 3^rd^ to 8^th^ grade^a^^,^ ^b^.

Difference in predicted literacy scores between		Grade					
ELGAN	TERM	3	4	5	6	7	8
LL	LH	-100.1	-105.4***	-79.1**	-102.3***	-90.0***	-85.7***
LL	HL	-125.7***	-99.5***	-83.9**	-85.2**	-105.4***	-73.0**
LL	HH	-195.6***	-202.4***	-189.7***	-192.2***	-170.8***	-167.9***
LH	LL	8.6	13.9	-12.4	10.8	-1.6	-5.8
LH	HL	-71.3*	-39.9	-50.6	-28.7	-61.2*	-33.1
LH	HH	-141.3***	-142.7***	-156.4***	-135.6***	-126.6***	-128.0***
HL	LL	34.2	8.0	-7.6	-6.3	13.9	-18.5
HL	LH	-20.2	-51.6	-41.0	-62.8*	-30.3	-58.4
HL	HH	-115.7***	-148.6***	-151.6***	-152.7***	-111.2***	-140.6***
HH	LL	104.1***	110.9***	98.2***	100.6***	79.3**	76.4**
HH	LH	49.8	51.2	64.9*	44.1	35.1	36.6
HH	HL	24.2	57.1	60.1*	61.2*	19.7	49.1

Abbreviations: ELGAN, extremely low gestation newborn; Social factors: LL–Low SES, Low Maternal Education; LH–Low SES, High Maternal Education; HL–High SES, Low Maternal Education; HH–High SES, High Maternal Education.

^a^Difference in predicted literacy scores: Predicted ELGAN literacy scores—predicted term literacy scores;

^b^Statistical significance are labeled as ***P <.001; **P <.01; *P <.05.

Otherwise, P-value was not significant (P ≥.05).

**Table 4:** Difference in predicted mathematics scores between ELGANs and term infants at different levels of maternal social factors^a^^,^^b^.

Difference in predicted mathematics scores between		All Grades
ELGAN	Term	
LL	LH	-78.7***
LL	HL	-69.3***
LL	HH	-130.0***
LH	LL	-17.9
LH	HL	-38.9*
LH	HH	-99.6***
HL	LL	-27.4
HL	LH	-57.8***
HL	HH	-109.1***
HH	LL	33.4*
HH	LH	2.9
HH	HL	12.4

Abbreviations: ELGAN, extremely low gestation newborn; Social factors: LL–Low SES, Low Maternal Education; LH–Low SES, High Maternal Education; HL–High SES, Low Maternal Education; HH–High SES, High Maternal Education.

^a^Difference in predicted mathematics scores: Predicted ELGAN mathematics scores—predicted Term mathematics scores.

^b^Statistical significance are labeled as ***P <.001; **P <.01; *P <.05; Otherwise, P value was not significant (P ≥.05).

The results in mathematics model were similar to the literacy model (Table 4). The difference in the predicted mathematics scores between advantaged (HH) ELGANs and disadvantaged (LL) term infants was significant and estimated at 33.4 points. However, in the mathematics model, ELGANs with either high SES (HL) or advanced education (LH) had significantly lower scores than term infants with either advanced education (LH) or high SES (HL).

## Discussion

This study examined the impact of GA and maternal SES and education level on literacy and mathematics achievement-test scores from grade 3 (early childhood) through grade 8 (early adolescence). While there were significant deficits in academic performance for ELGANs compared to PT, LPT, and term infants, the deficit could be offset by higher SES and better-educated mothers. These favorable social factors were critical to a child’s academic achievement. While their impact modestly diminished over time for literacy, it remained constant across grades for mathematics.

There is one previously published study that explicitly evaluated the relative influence of prematurity and social factors on academic performance, though the authors used aptitude tests (Bayley Scales of Infant Development and Wide Range Achievement Test) [28]. They compared aptitude test results at 8 and 18 years of age of former ELGANs and normal-weighted infants and concluded that extreme prematurity rather than social factors imprinted academic performance [28]. On the other hand, our study used curriculum-based standardized achievement tests rather than aptitude tests and concluded that the influence of favorable maternal social factors was more significant than the degree of prematurity on test scores.

Preterm infants of various GAs [29] have impaired brain size and maturation at term despite the absence of severe brain injury. Nevertheless, the neonatal brain is very sensitive to environmental exposures and can be molded as it matures over the first 2 years of life [30,31]. Substantial brain growth and significant increase in the number of synapses occur in infancy and early childhood [31–34]. Both environmental deprivation and enrichment can then drastically shape the brain [31–36]. Parental education and family income significantly impact brain growth, particularly in the earlier years of childhood [37]. In fact, our analysis showed that the influence of favorable maternal social factors on achievement-test scores was greater in 3^rd^ grade than 4^th^-8^th^ grades.

Previous studies described poor performance of preterm infants on mathematics skills [38,39]. Likewise, our analysis showed a significant impact of prematurity on mathematics test scores. The difficulties in mathematics were previously attributed to injuries in specific regional brain areas and underdeveloped gray matter in the left parietal lobe of preterm infants [38,39].

Consistent with previous work [40], we observed that male infants performed worse than female on achievement-test scores. A meta-analysis on gender disparities in scholastic achievement reported a large female advantage in language courses and a modest gain in mathematic scores [40]. The gender gaps were attributed to a myriad of factors including differences in learning styles [40]. In our model, black infants performed worse than other races on standardized tests. These infants, as well, were more likely to have disadvantaged mothers than other races. Previous studies supported our results as they evaluated the socioeconomic disparities and educational gaps across races in the U.S. and showed that black infants are more likely to attend school with lower academic performance [41]. Blacks are typically overrepresented among ELGANs in the U.S. [42]. Thus, the overall burden of lower social factors in ELGANs could have disproportionately impacted our results. Interestingly, however, in our cohort, the proportion of white and black ELGANs with LL was similar. In addition, our study population included a very small percentage (18/1424, 1.3%) of infants with severe brain injury (grades 3–4 intraventricular hemorrhage, periventricular leukomalacia, and/or ventriculomegaly). Consequently, we were unable to detect the impact of this variable on achievement-test scores.

Our study had multiple strengths, most notably the longitudinal evaluation using a curriculum-based measure of academic performance over a 6-year period from childhood through early adolescence, a study cohort that included infants of all GAs, and an evaluation of the contribution of prematurity and maternal social factors to achievement-test scores. There remain certain limitations. Our analysis was a retrospective and not a prospective assessment. However, other studies found associations between fetal and infant parameters and adolescent or adult performance or diseases and did not account for intervening childhood influences such as household characteristics or environmental influences [43]. In addition, we had incomplete paternal education data and used insurance status rather than household income or census-tract data on poverty, as a proxy for SES. However, insurance status was previously identified, as a critical predictor of neonatal outcomes and a commonly reported proxy for SES in outcome studies of preterm infants in the U.S. [4]. We were also unable to gather data on parental interaction with their children or the home environment. In addition, we used units of prematurity and maternal social factors that were artificially categorized and thus could have impacted our results. However, the separation of infants into GA groups, and our dichotomous classification of SES and education level were not arbitrary, as these categorizations have been widely used in the literature [4,21–23]. Thus, we believe that alternate categorizations would lead to similar conclusions. We also lacked data on specific perinatal risk factors such as length of ventilation and hospital readmissions and the quality of the schools the children attended. Despite these limitations, we consistently observed that the impact of social factors on achievement-test scores seemed to outweigh the influence of prematurity.

## Conclusion

The academic performance of preterm and term children is determined by a complex interaction of biological, genetic, environmental, and social factors [4]. Our study specifically addressed the role of prematurity and maternal social factors and established that maternal social factors seem to have an enduring influence on children’s academic performance over time (at least up to 8^th^ grade). Favorable social factors could possible overcome the adverse effects of prematurity, perhaps due to more stimulating home environments and higher parental expectations. The role of social factors must be considered in future studies evaluating the impact of prematurity on outcomes.

## Supporting information

S1 Table Distribution of gestational-age groups and maternal social factors among black children. (DOCX)

S2 Table Distribution of gestational-age groups and maternal social factors among white children. (DOCX)

S3 Table Test score availability from 3^rd^ to 8^th^ grade. (DOCX)

S4 Table Achievement-test scores by gestational-age groups and grade level. (DOCX)

S5 Table Univariate association between predictor variables and literacy and mathematics scores. (DOCX)
