# Supplementary material for: The impact of prematurity and maternal socioeconomic status and education level on achievement-test scores up to 8th grade
Source: PLoS One. 2018 May 31;13(5):e0198083. doi: 10.1371/journal.pone.0198083 (PMC5978790; doi:10.1371/journal.pone.0198083)
Supplement: S1 Table — (DOCX) [file pone.0198083.s001.docx]

**S1 Table. Distribution of gestational-age groups and maternal social factors among black children**

| Variable | LL (n=449) | LH (n=113) | HL (n=28) | HH (n=28) |
| --- | --- | --- | --- | --- |
| ELGAN, n (%) | 17 (3.8) | 9 (8.0) | 4 (14.2) | 0 (0) |
| PT, n (%) | 45 (10.0) | 11(9.7) | 2 (7.1) | 1 (3.5) |
| LPT, n (%) | 68 (15.1) | 16 (14.2) | 5 (17.6) | 4 (14.2) |
| Term, n (%) | 319 (71.0) | 77 (68.1) | 17 (60.7) | 23 (82.1) |

^a^The proportion of black infants who had mothers with social strata (LL): 449/618=72.6%.

^b^Among ELGAN (n=30), 17/30=56.7% had mothers with social strata (LL); among term infants (n=436), 319/436=73.1 % had mothers with social strata (LL).

Abbreviations: ELGAN, extremely low gestation newborn; LPT, late preterm; PT, preterm; Maternal Social factors: LL–Low SES, Low Maternal Education; LH–Low SES, High Maternal Education; HL–High SES, Low Maternal Education; HH–High SES, High Maternal Education.
