# Supplementary material for: The impact of prematurity and maternal socioeconomic status and education level on achievement-test scores up to 8th grade
Source: PLoS One. 2018 May 31;13(5):e0198083. doi: 10.1371/journal.pone.0198083 (PMC5978790; doi:10.1371/journal.pone.0198083)
Supplement: S2 Table — (DOCX) [file pone.0198083.s002.docx]

**S2 Table. Distribution of gestational-age groups and maternal social factors among white children**

| Variable | LL^a^ (n=407) | LH (n=63) | HL (n=62) | HH (n=127) |
| --- | --- | --- | --- | --- |
| ELGAN^b^, n (%) | 14 (3.4) | 2 (3.2) | 3 (4.8) | 9 (7.0) |
| PT, n (%) | 57 (14.0) | 9 (14.3) | 18 (29) | 23 (18.1) |
| LPT, n (%) | 67 (16.4) | 8 (12.7) | 8 (12.9) | 21 (16.5) |
| Term, n (%) | 269 (66.1) | 44 (69.8) | 33 (53.2) | 74 (58.3) |

^a^The proportion of white infants who had mothers with social strata (LL): 407/659=61.7%.

^b^Among ELGAN (n=28), 14/28=50% had mothers with social strata (LL); among term infants (n=420), 269/420=64.0% had mothers with social strata (LL).

Abbreviations: ELGAN, extremely low gestation newborn; LPT, late preterm; PT, preterm; Maternal Social factors: LL–Low SES, Low Maternal Education; LH–Low SES, High Maternal Education; HL–High SES, Low Maternal Education; HH–High SES, High Maternal Education.
