# Supplementary material for: The impact of prematurity and maternal socioeconomic status and education level on achievement-test scores up to 8th grade
Source: PLoS One. 2018 May 31;13(5):e0198083. doi: 10.1371/journal.pone.0198083 (PMC5978790; doi:10.1371/journal.pone.0198083)
Supplement: S3 Table — (DOCX) [file pone.0198083.s003.docx]

**S3 Table. Test score availability from 3^rd^ to 8^th^ grade**

|  | **ELGAN** | **PT** | **LPT** | **TERM** | **Total** |
| --- | --- | --- | --- | --- | --- |
|  | (*n* = 58) | (*n* = 171) | (*n* = 228) | (*n* = 967) | (*n* = 1424) |
| **Literacy score availability**, n (%) | | | | |  |
| Grade 3 | 55 (94.8) | 158 (92.4) | 203 (89.0) | 860 (88.9) | 1276 (89.6) |
| Grade 4 | 53 (91.4) | 161 (94.2) | 203 (89.0) | 891 (92.1) | 1308 (91.8) |
| Grade 5 | 56 (96.6) | 158 (92.4) | 212 (93.0) | 893 (92.3) | 1319 (92.6) |
| Grade 6 | 54 (93.1) | 159 (93.0) | 208 (91.2) | 896 (92.7) | 1317 (92.4) |
| Grade 7 | 52 (89.7) | 158 (92.4) | 206 (90.4) | 881 (91.1) | 1297 (91.0) |
| Grade 8 | 45 (77.6) | 142 (83.0) | 190 (83.3) | 811 (83.9) | 1188 (83.4) |
| **Mathematics score availability**, n (%) | | | | |  |
| Grade 3 | 55 (94.8) | 158 (92.4) | 203 (89.0) | 860 (88.9) | 1276 (89.6) |
| Grade 4 | 53 (91.4) | 161 (94.2) | 203 (89.0) | 891 (92.1) | 1308 (91.8) |
| Grade 5 | 56 (96.6) | 158 (92.4) | 212 (93.0) | 893 (92.3) | 1319 (92.6) |
| Grade 6 | 54 (93.1) | 159 (93.0) | 210 (92.1) | 898 (92.9) | 1321 (92.7) |
| Grade 7 | 52 (89.7) | 156 (91.2) | 206 (90.4) | 873 (90.3) | 1287 (90.3) |
| Grade 8 | 44 (75.9) | 140 (81.9) | 189 (82.9) | 799 (82.6) | 1172 (82.3) |

Abbreviations: ELGAN, extremely low gestation newborn; LPT, late preterm; PT, preterm
