# Supplementary material for: The impact of prematurity and maternal socioeconomic status and education level on achievement-test scores up to 8th grade
Source: PLoS One. 2018 May 31;13(5):e0198083. doi: 10.1371/journal.pone.0198083 (PMC5978790; doi:10.1371/journal.pone.0198083)
Supplement: S4 Table — (DOCX) [file pone.0198083.s004.docx]

**S4 Table. Achievement-test scores by gestational-age groups and grade level**

|  | Proficiency  Threshold^a^ | ELGAN | PT | LPT | TERM | p |
| --- | --- | --- | --- | --- | --- | --- |
|  |  | (*n* = 58) | (*n* = 171) | (*n* = 228) | (*n* = 967) |  |
| **Literacy**^b^ | | | | | | |
| Grade 3 | 499 | 424.83 (94.89) | 476.68 (89.61) | 472.52 (85.43) | 485.37  (80.45) | <.0001 |
| Grade 4 | 558 | 527.01  (97.7) | 579.94  (90.3) | 578.49 (85.97) | 590.84  (81.46) | <.0001 |
| Grade 5 | 603 | 576.2  (92.88) | 627.41 (87.48) | 626.11 (83.73) | 637.02  (78.55) | <.0001 |
| Grade 6 | 640 | 610.14 (93.67) | 660.65 (85.43) | 663.88 (83.63) | 673.65  (79.34) | <.0001 |
| Grade 7 | 672 | 655.25 (89.59) | 701.38  (79.6) | 705.13 (79.44) | 716.72  (75.72) | <.0001 |
| Grade 8 | 699 | 713.44 (93.66) | 765.36 (76.53) | 768.35 (76.02) | 779.03  (75.06) | <.0001 |
| **Mathematics**^b^ | | | | | | |
| Grade 3 | 499 | 477.41 (52.85) | 528.77 (48.23) | 527.01 (46.78) | 536.78  (43.05) | <.0001 |
| Grade 4 | 558 | 533.82 (53.59) | 585.06 (48.94) | 584.41 (47.32) | 594.67  (43.24) | <.0001 |
| Grade 5 | 603 | 568.13 (52.11) | 617.34  (49.4) | 616.44 (47.26) | 625.82  (43.53) | <.0001 |
| Grade 6 | 640 | 616.36 (52.77) | 665.96 (48.09) | 667.13 (47.51) | 675.63  (43.76) | <.0001 |
| Grade 7 | 672 | 646.5  (53.71) | 693.35 (47.03) | 696.41 (46.96) | 705.65  (43.57) | <.0001 |
| Grade 8 | 699 | 656.18 (56.29) | 703.85 (46.73) | 705.33 (46.33) | 714.06  (44.27) | <.0001 |

Abbreviations: ELGAN, extremely low gestation newborn; LPT, late preterm; PT, preterm

^a^Proficient or advanced scores (proficient) represent performance at or above grade level,

while basic and below basic scores (non-proficient) represent performance below grade level

^b^Data expressed as mean (standard deviation)
