# Supplementary material for: The impact of prematurity and maternal socioeconomic status and education level on achievement-test scores up to 8th grade
Source: PLoS One. 2018 May 31;13(5):e0198083. doi: 10.1371/journal.pone.0198083 (PMC5978790; doi:10.1371/journal.pone.0198083)
Supplement: S5 Table — (DOCX) [file pone.0198083.s005.docx]

**S5 Table. Univariate association between predictor variables and literacy and mathematics scores**

| Predictor Variable | Literacy Model | | | Mathematics Model | | |
| --- | --- | --- | --- | --- | --- | --- |
|  | Coefficient | 95% CI | P | Coefficient | 95% CI | P |
| **Grade** | 53.7 | (52.3, 55.1) | <0.001 | 36.4 | (35.6, 37.1) | <0.001 |
| **Birth weight** | 0.01 | ( 0.00, 0.02) | 0.004 | 0.02 | ( 0.01, 0.02) | <0.001 |
| **Gestational age** |  |  | 0.03 |  |  | <0.001 |
| TERM | Ref | Ref |  | Ref | Ref |  |
| LPT | -9.3 | (-33.3, 14.7) |  | -8.9 | (-22.3, 4.5) |  |
| PT | -10.2 | (-37.2, 16.8) |  | -7.4 | (-22.5, 7.7) |  |
| ELGAN | -66.0 | (-110.1, -21.8) |  | -58.7 | (-83.4, -34.0) |  |
| **Size at birth** |  |  | 0.544 |  |  | 0.043 |
| AGA | Ref | Ref |  |  |  |  |
| LGA | 9.7 | (-25.3, 44.8) |  | 14.2 | (-5.5, 33.9) |  |
| SGA | -11.6 | (-36.9, 13.7) |  | -13.9 | (-28.1, 0.4) |  |
| **Race** |  |  | <0.001 |  |  | <0.001 |
| Black | Ref | Ref |  | Ref | Ref |  |
| White | 102.2 | (85.3, 119.1) |  | 69.1 | (59.8, 78.4) |  |
| Other | 85.8 | (48.4, 123.2) |  | 67.9 | (47.3, 88.5) |  |
| **Male** | -81.4 | (-98.2, -64.7) | <0.001 | -13.5 | (-23.2, -3.8) | 0.007 |
| **Multiple gestation** | -35.4 | (-71.8, 1.0) | 0.057 | -27.9 | (-48.4, -7.5) | 0.007 |
| **Vaginal route** | 0.5 | (-18.1, 19.2) | 0.955 | 2.8 | (-7.7, 13.3) | 0.604 |
| **Apgar 1 minute ≥7** | 44.7 | (23.9, 65.5) | <0.001 | 26.4 | (14.8, 38.1) | <0.001 |
| **Apgar 5 minutes ≥7** | 30.5 | (-3.9, 65.0) | 0.083 | 27.1 | (7.6, 46.5) | 0.006 |
| **Meconium** | -34.6 | (-63.9, -5.2) | 0.021 | -19.9 | (-36.4, -3.4) | 0.018 |
| **Length of stay** | -1.0 | (-1.5, -0.5) | <0.001 | -0.7 | (-1.0, -0.5) | <0.001 |
| **Brain injury** |  |  | 0.594 |  |  | 0.095 |
| None | Ref | Ref |  | Ref | Ref |  |
| Mild | -34.3 | (-101.3, 32.7) |  | -30.1 | (-67.7, 7.6) |  |
| Severe | 6.8 | (-70.9, 84.4) |  | -33.8 | (-77.3, 9.8) |  |
| **Maternal age** |  |  | <0.001 |  |  | <0.001 |
| <20 | Ref | Ref |  | Ref | Ref |  |
| 20-29 | 1.7 | (-19.1, 22.4) |  | 7.2 | (-4.5, 18.8) |  |
| 30-39 | 58.4 | (30.5, 86.2) |  | 35.1 | (19.4, 50.7) |  |
| ≥40 | 68.5 | ( 5.2, 131.7) |  | 38.6 | (2.9, 74.2) |  |
| **Gravidity** |  |  | 0.003 |  |  | 0.002 |
| 1 | Ref | Ref |  | Ref | Ref |  |
| 2-3 | -18.5 | (-38.0, 0.9) |  | -8.9 | (-19.8, 2.1) |  |
| >3 | -42.5 | (-67.2, -17.8) |  | -24.8 | (-38.7, -10.9) |  |
| **Maternal diabetes** |  |  | 0.093 |  |  | 0.037 |
| None | Ref | Ref |  |  |  |  |
| Pre-pregnancy | -55.5 | (-111.9, 1.0) |  | -34.8 | (-66.2, -3.4) |  |
| Gestational | 26.8 | (-27.1, 80.6) |  | 20.3 | (-10.0, 50.6) |  |
| **PIH** | -12.9 | (-34.6, 8.6) | 0.239 | -3.2 | (-15.3, 9.0) | 0.609 |
| **PROM** | -28.6 | (-53.8, -3.4) | 0.026 | -18.4 | (-32.6, -4.3) | 0.011 |
| **Chorioamnionitis** | -5.5 | (-46.7, 35.7) | 0.795 | -6.3 | (-29.6, 16.9) | 0.593 |
| **Prenatal care** | 29.8 | (-22.2, 81.7) | 0.261 | 35.4 | (6.3, 64.5) | 0.017 |
| **Smoking** | -2.2 | (-58.8, 54.4) | 0.939 | -8.8 | (-40.6, 23.1) | 0.589 |
| **Substance abuse** | -42.3 | (-101.9, 17.3) | 0.165 | -37.5 | (-71.0, -4.0) | 0.028 |
| **Social factors** |  |  | <0.001 |  |  | <0.001 |
| LL | Ref | Ref |  | Ref | Ref |  |
| LH | 31.6 | (6.6, 56.6) |  | 20.6 | (6.6, 34.6) |  |
| HL | 52.4 | (19.4, 85.5) |  | 26.7 | (8.2, 45.2) |  |
| HH | 159.0 | (132.4, 185.7) |  | 94.7 | (79.8, 109.6) |  |

Abbreviations: AGA, appropriate for gestational age; CI, confidence interval; ELGAN, extremely low gestation newborn; LGA, large for gestational age; LPT, late preterm; PIH, pregnancy-induced hypertension; PROM, prolonged rupture of membranes; PT, preterm; Ref, reference; SGA, small for gestational age; Social factors: LL–Low SES, Low Maternal Education; LH–Low SES, High Maternal Education; HL–High SES, Low Maternal Education; HH–High SES, High Maternal Education
